# The Role of the Psycho-Oncologist during the COVID-19 Pandemic: A Clinical Breast Cancer Case Report

**DOI:** 10.3390/bs12070211

**Published:** 2022-06-25

**Authors:** Giulia Silvestri, Caterina Borgese, Samuela Sommacal, Letizia Iannopollo, Grazia Cristaldi, Samantha Serpentini

**Affiliations:** Veneto Institute of Oncology IOV—IRCCS, 35128 Padua, Italy; giulia.silvestri@iov.veneto.it (G.S.); caterina.borgese@studenti.unipd.it (C.B.); samuela.sommacal@iov.veneto.it (S.S.); letizia.iannopollo@iov.veneto.it (L.I.); grazia.cristaldi@gmail.com (G.C.)

**Keywords:** breast cancer, COVID-19, well-being, quality of life, social support, distress, supportive care

## Abstract

The 2019 coronavirus pandemic (COVID-19) has been very stressful, but more so for those with cancer. Patients with cancer experienced more pandemic-related stress and psychological distress than those without a cancer diagnosis. This case report, about a breast cancer patient, is presented in order to emphasize (1) the importance of the management of psychological care in oncology, (2) the need for a thorough understanding of the efficacy of the role of the psycho-oncologist and related interventions in a breast care unit for the health of both patients and professionals to improve clinical outcomes, and (3) the emerging health concerns of breast cancer patients in the context of the COVID-19 pandemic.

## 1. Introduction

Patients suffering from cancer reported experiencing significantly more stress linked to the COVID-19 pandemic as well as greater psychological symptoms compared to those without cancer [1]. 

In relation to the reorganization of patients during the COVID-19 pandemic, it is useful to divide them into two groups: patients who have completed the therapeutic path (off-therapy) and those who are still being treated. In this case, the guidelines of the Ministry of Health state the following:

- Local health services identify and prepare paths and spaces dedicated to the oncological patients;
- Healthcare professionals wear appropriate personal protective equipment;
- Identify specific strategies to already guarantee the diagnostic-therapeutic path initiated for patients in case of potential exposure of a cancer patient to subjects infected with SARS-CoV-2 [2].

During the COVID-19 period, cancer patients experienced clinically significant levels of psychological distress (i.e., anxious and depressive symptoms), pain, and insomnia [3,4,5,6]. In addition, immunosuppression associated with cancer treatments (e.g., chemotherapy) resulted in a greater risk of infection of COVID-19 for oncological patients in treatment compared to the general population [3]. The increased vulnerability to COVID-19 and restrictions imposed by the pandemic, such as lockdowns and physical distancing, affected both access to cancer care [7,8] and social support, resulting in increased levels of distress [9]. Thus, these vulnerabilities in patients with cancer brought on by COVID-19 resulted in anxiety and depression levels that exceeded even the typically elevated levels of patients suffering from cancer compared to the general population prior to the pandemic [10].

Elevated distress levels of patients with cancer compared to the general population have been attributed to the diagnosis itself, which can be more or less serious, including treatments such as surgery, radiation therapy, chemotherapy, and hormone therapy, which are often destructive and harmful to patients’ body image and cause disruptions in relationships and work as well as variation in their ability to cope with stress. Research has shown that, prior to the COVID-19 pandemic, about 30–40% of breast cancer patients suffered from depression, anxiety, or adjustment disorders [3,7,10], and the incidence has been steady during the pandemic. In 30 countries, a meta-analysis of 72 studies showed that the prevalence of depression in patients with breast cancer was 32.2% [11]. In addition, living alone, comorbidity, late-stage diagnosis, and spread of the disease are risk factors that exacerbate depression, anxiety, and insomnia [12]. Finally, as noted earlier, during the COVID-19 lockdowns, it was difficult to preserve social relationships and social support, which also impacted well-being and quality of life at a time when social support can be an offset to distress.

Therefore, it is critical to assess the psychological symptoms in patients suffering from cancer in order to mitigate symptoms and increase their quality of life. In the context of the COVID-19 pandemic, the goal of diagnosing and treating anxiety and depression is also to optimize the effects of medical treatments and adherence to medical regimens. 

The role of the psycho-oncologist is to support the cancer patient in accepting the disease, searching for psychological and existential meaning [13], and treating anxiety and depression symptoms [14]. 

Compared to cancer patients under treatment, a recent Italian study [15] reports the following psychological reactions:

- Sense of vulnerability;
- Sense of helplessness and physical suffering associated with COVID-19 that can cause generalized fear and anxiety;
- Sense of loneliness, increased by constraints on family members, with a possible reduction of adherence to therapy;
- Experience of dependence on therapies, with consequent fear of not receiving adequate treatment;
- Perception of responsibility for the risk of contagion;
- Fear of physical contact with health professionals.
  Patients and caregivers experience the following:
- Intensification of psychological distress, in particular, increased anxiety symptoms;
- Existential and psychological uncertainty, with consequent demoralization;
- Decrease in self-efficacy and confidence in one’s coping strategies with the disease.

Thus, there are a variety of psychological interventions available to help the patient to manage the therapeutic treatments as well as the psychological symptoms caused by cancer and its treatments [16].

Furthermore, it is important to make consideration with respect to health professionals: COVID-19 also increased symptoms of anxiety, psychological distress, emotional burden, clinically significant depression, and a higher perceived risk of infection, leading to cognitive, emotional, and behavioral disturbances [17], for healthcare workers in intensive care units [18].

It is fundamentally important to pursue the following objectives when intervening in the lives of patients with cancer: foster a recognition of one’s own needs, help process trauma, help to rebuild body image, and shift attention to individual (coping strategies, behaviors) and social resources [12,19]. Moreover, the role and expertise of the psycho-oncologist is now formally recognized within the context of medical centers that focus on breast cancer [19,20,21].

The purpose of this case report is to emphasize the importance and value added by psychologists in medical oncology care and, in particular, to describe the critical role of psychological interventions and support in typical breast care service during the COVID-19. Moreover, this psychological management has the following aims:

- Prevent and contain the fear of contagion, which can compromise the management of cancer treatments and patient adherence to them;
- Identify and manage the level of psychological distress and related symptoms
  (anxiety, depression, insomnia, fatigue, etc.) experienced by patients and their family in this emergency situation;
- Promote the expression of emotions;
- Increase active coping strategies, self-efficacy/empowerment attitudes, and better adaptation to the situation;
- Support healthy lifestyles and precautionary behaviors;
- Provide correct information to patients and make known the support systems available.

## 2. Case Report 

Sofia (pseudonym) is a 40-year-old female who is married and has two children as well as a supportive family and social network. She has no personal or family history of psychiatric illnesses and had no other comorbid diseases. In November of 2019, she noted swelling and a sensation of heat in her left breast. Then in June of 2020, she felt focal thickening in the upper-external left quadrant. Shortly after that, in July of 2020, Sophia scheduled her first consultation at the Cancer Institute in Padova, at which time imaging showed the presence of a heteroplastic lesion in a large part of the upper-external quadrant. On her first visit to the oncologist in August 2020, the diagnosis was infiltrating breast cancer. The patient was in good general condition and concurred with the oncologist that treatment should be initiated. The treatment involves a series of chemotherapy cycles in association with hormonal therapy. As for the follow-up, the patient will also perform radiotherapy sessions. At the end of the treatment, for the purpose of prognosis, the oncologist will perform specific tests aimed at identifying the disease state.

During adjuvant chemotherapy treatment, on the recommendation of the oncologist, Sofia asked for a psychological consultation. In January 2021, Sophia also had mastectomy and axillary lymphadenectomy surgery. The last operations were performed in September. Then Sofia underwent a mastectomy and status post six-cycle chemotherapy because of the presence of a tumor on her left breast.

Our approach, as a strategic plan, was patient-centered: it was based on therapeutic strategies, on the evaluation of symptoms and side effects of therapies [22], on a correct analysis of the quality of life, and on the fundamental detection of unmet needs [23].

The pattern of sessions included an initial intervention with the whole family and some only with Sophia. 

Cancer has the potential to transform the family system of the development of adaptive processes connected to the dynamic equilibrium of the patient-family-care team system. 

The disease breaks family homeostasis, and it is, therefore, possible to evaluate the oncological sickness not only as an individual’s illness but also as a family disease. Distress, burden, and strain, related to cancer, are the main indicators of the psycho-social impacts on family members [24]. 

It has been shown that there is an important reciprocity of emotional influence with the patient: when the latter presents a psychic disorder (anxiety or depression), the caregiver shows an increased risk of developing a similar emotional disorder [25,26]. 

Family support aims to promote adaptive coping, experiencing the disease event more positively in the family context; therefore, this maintains more positive coping from the patient [27].

We have always proceeded towards the promotion of self-efficacy and empowerment strategies with the aim of encouraging active and functional coping methods for adaptation, improving future planning. 

In the first session, Sofia came to the psycho-oncology clinic with her family, husband, and children (a 13-year-old son and an 8-year-old daughter). The request she made was for family and individual support for the management of emotional experiences related to the illness and related treatments. The patient and the family members narrated their experience of the disease, from the moment of diagnosis to the current day, focusing on their emotional experiences over that period of time. 

Later, family support was conducted through telehealth sessions in order to process the relational dynamics and promote familial support and active coping strategies for managing the emotional experience. 

The following sessions were conducted with only Sophia, focusing on the management of the disease and her emotional reactions, particularly in the context of the COVID-19 pandemic. Thus, the therapeutic process included emotional (trauma) processing as well as building support for the patient by analyzing and fostering positive family dynamics and also promoting active, agentic coping strategies. 

Sophia presented psychological symptoms such as distress, depression, and anxiety related to the loss of a sense of control in the management of roles and self-image in the transformation of planning and short- and long-term aims. She also reported distress focused on fear and the trauma of the diagnosis. In this first phase of treatment, the defense expressed by Sofia was denial. She was conversant, but the clinician observed mood swings, emotional changes, and the absence of a positive attitude. Sofia reported significant physical symptoms, such as fatigue, weakness, fragility, insomnia, dyspnea, pain, and a lack of appetite. The patient appeared to be exhausted by the therapy process as she shared negative emotional experiences related to having cancer and concerns for her children. Sophia reported significant distress as a result of the COVID-19 pandemic situation, during which she experienced greater isolation even within the family, including isolation from her children at times to reduce the risk of COVID-19 infection. During this period of greater isolation, psychological support was important to sustain in therapy and in the family to help mitigate Sophia’s tendency to focus on the feeling of emotional loneliness linked to the uncertainty of a global health emergency. 

Due to COVID-19, specific strategies were performed, such as relaxation techniques that are able to contribute to the management, control, and containment of physical and psychological symptoms. In fact, during some sessions, body mediation techniques were performed, such as autogenic training, for global therapeutic purposes (e.g., distress, anxiety, pain, side effects of the therapy). Schultz defines them as “a series of rational physiological exercises, intended to put the body and the psyche at rest” [23,28] (pp. 137–143).

Psychosocial intervention grew the awareness of psychological problems associated with cancer, and its treatment led to the development of supportive interventions for patients and their families, promoting quality of life (QoL) and well-being. 

Sofia began to adapt and maintain good psycho-emotional relaxation, supported by the coping strategies that gradually appeared during the psychological path. The outcomes of the psychotherapeutic treatments increased an adequate internal locus of control in the psychological adaptation to the oncological disease, favoring combative coping in the patient and promoting a proactive experience towards the disease, which was experienced as a challenge to be actively faced. The improvements due to the therapies allowed the patient to learn to have greater confidence and manage the discomforts related to the treatments, which are experienced with optimism and a better level of adaptation. The patient developed the skill of managing anxiety about the future and the fear of recurrence in an adjustment and functional way in the pandemic context.

## 3. Discussion

Neoplastic pathology can have profound repercussions on the psychological setting of the patient and her family, negatively affecting quality of life, adherence to medical care, the perception of side effects, the doctor–patient relationship, and the duration of hospitalization and rehabilitation.

The impact of cancer affects many dimensions of a person’s existence: physical, psychological, social, and spiritual [29]. One-third of patients experience distress, anxiety, depression, adjustment problems, post-traumatic symptoms, and sleep disorders [30,31]. Many of these patients develop a psychiatric diagnosis, such as anxiety (10.3%), depression (24.6%), and adjustment disorders (19.4%) [31].

Different scientific evidence indicates that people with serious or critical health conditions, including cancer, due to their immunosuppressive status, experience an increased risk of contagion to COVID-19 and any related complications [32].

The condition of greater “vulnerability” establishes in patients an increase in psychological symptoms such as anxiety, anger, anguish, fear, and a sense of loneliness [33]. 

Furthermore, the pandemic has created the need to combat the infection by imposing social distancing and numerous health restrictions that affect access to places of care and the regular use of oncological therapies [34,35].

This circumstance causes concern about treatment for their disease [35]. Patients experience ambivalent psychological emotions: on the one hand, the worry of “life-saving” therapies and, on the other, the fear of exposing oneself to the risk of contagion during treatments [15].

Returning to our clinical case, the cure and treatment of carcinoma are approached with a multidisciplinary approach with adequate attention to the physical, psychological, self-efficacy, and well-being of the patient as well as their own social network to increase the best adaptation strategies to the disease. Sofia, once she has overcome the initial shock of the disease, has to live with COVID-19, which bursts and complicates her therapeutic path, making it even more difficult to distance herself from her family and her social network: experiences of uncertainty and loneliness that arise and increase exponentially. The event of illness in a pandemic context requires an important path of adapting to the disease deriving from the COVID-19 pandemic, restoring psycho-physical balance and strengthening. 

Counseling represents the first step in taking care of patients and their families through an initial emotional, psychological, and cognitive assessment related to the management of the critical situation. This allows a first clinical picture of psycho-social needs and the level of psycho-emotional distress experienced, deepening the internal psychic resources and the presence of any internal coping methods related to the lived experience of the disease. The consultancy aims to capture, analyze, and elaborate doubts, perplexities, and expectations in order to promote adaptation by encouraging the integration of the disease event within the patient’s personal life story [21,36].

Feeling depression, anxiety, and fear is very common, and it is a normal response to one’s life-changing experience [37]. People with cancer feel distressed about it at any time after a cancer diagnosis. As the cancer situation changes, they all must cope with new stressors as well as with the old, and their feelings often change, too. 

In the subsequent interviews, psycho-oncological support aims to deepen the existential meanings experienced and expressed by the patient and the family.

The emotions of concern, fear, and disorientation are related to the diagnosis and the disease path. Patients may be afraid of uncontrolled pain and death, including what might happen to loved ones. It is important to share these feelings and fears.

The importance of social support, even for the patient’s family, is proof of this. In fact, it is observed that patients with greater social support tend to feel less anxious and depressed and report better quality of life, helping with cancer management.

Depression and sickness cause great distress, impair functioning, and can even make the patient less able to follow the cancer treatment plan [36].

During the treatment phase, the psycho-oncologist validates the patient’s experience, elaborating emotional problems in order to bring out the appropriate coping modalities, which favor adjustment and adherence to the therapeutic path.

During hospitalization, the psycho-oncologist has an important role in the pain, which impacts quality of life. The clinician encourages adequate management strategies and promotes communication between the patient and care team; this increases compliance and favors the treatment path’s adherence [21].

Each disease and treatment phase has internal and specific aspects. This opens up a new clinical context, accompanying patients through the path of care. The psycho-oncologist processes emotions, increasing communication with the family and supporting the patient’s social network. Oncological patients often express feelings of despair, loss of meaning, uncertainty and helplessness, pessimism, and difficulty in adapting, which underline the presence of an affective state of demoralization. Consequently, psycho-oncological support is focused on the therapy’s treatment of cancer-related symptoms (e.g., hair loss, fatigue, inappetence, insomnia), and it promotes adjustment linked to the specific disease stage. 

Sofia’s case explicitly deals with hospitalization (inpatient) and the outpatient clinic.

Finally, the role of the psychologist is also very important during the end-stage of the disease, where perspectives are based on a greater awareness of the personal relationship with life and death.

In this phase, the clinician promotes the possibility of human and professional growth through the path of approaching the most intimate part of the personal meaning of life.

Death education teaches patients to become more aware and sensitive companions to themselves and those around them on the difficult path of death. In this phase, the patient is invested with anxieties and questions that require sensitivity, attention, and even human competence. The psycho-oncologist endorses patients’ disorientation, preparing the person for the prospect of “letting go”. This attitude is a very useful approach because it supports the person in the death process towards a peaceful welcome [36].

## 4. Conclusions

Sofia’s case demonstrates the experience of a traumatic event, such as cancer, during the pandemic, causing severe rejection, non-acceptance, and destabilization.

Sofia’s story illustrates the psychological, existential, and social complexity of the experience of a young breast oncological patient during the particular historical context of COVID-19. The findings of the present case report suggest that psychological support is essential at every stage of the disease on the path of care in order to relieve depressive symptoms and distress and strengthen coping strategies. 

## Data Availability

Not applicable.
